# *Toxoplasma gondii* KCR is a Noncanonical Modulator of CSF2 Signaling that Targets the CSF2Rα–JAK2/STAT5 Axis

**DOI:** 10.1155/tbed/8426765

**Published:** 2026-05-09

**Authors:** Jie Mei, Xianglin Pu, Mengkai Zhang, Yue Liu, Chuang Meng, Lixin Xu, Xiaokai Song, Ruofeng Yan, Xiangrui Li, Mingmin Lu

**Affiliations:** ^1^ Ministry of Education (MOE) Joint International Research Laboratory of Animal Health and Food Safety, College of Veterinary Medicine, Nanjing Agricultural University, Nanjing, 210095, China, njau.edu.cn; ^2^ Jiangsu Key Laboratory of Zoonosis, Yangzhou University, Yangzhou, Jiangsu, 225009, China, yzu.edu.cn

**Keywords:** CSF2, immune evasion, JAK2/STAT5 signaling, KCR, *Toxoplasma gondii*

## Abstract

*Toxoplasma gondii* establishes infection in hosts by deploying effector proteins that reprogram cytokine‐driven immunity. While protozoan parasite‐encoded macrophage migration inhibitory factor (MIF) homologs exemplify cytokine mimicry, whether *T. gondii* targets additional host cytokine axes remains unclear. Here, we identify a *T. gondii* 3‐ketoacyl‐CoA reductase (KCR) as a noncanonical cytokine modulator that engages the granulocyte‐macrophage colony‐stimulating factor (CSF2) receptor alpha chain (CSF2Rα). In HEK293T cells, forward and reverse co‐immunoprecipitation (Co‐IP) assays demonstrated a specific interaction between KCR and murine CSF2Rα. The recombinant KCR triggered rapid activation of canonical CSF2 signaling in RAW264.7 macrophages, inducing phosphorylation of JAK2 and STAT5 to levels comparable to murine CSF2. Functionally, KCR enhanced NADPH oxidase‐dependent oxidative responses, increasing intracellular reactive oxygen species (ROS) and upregulating transcripts encoding core oxidase subunits, including CYBB, CYBA, NCF1, and NCF2. KCR also promoted phagocytic capacity, elevating FITC‐dextran uptake and inducing expression of complement, Fc, and scavenger receptor genes, including CR3, CD16, CD64, MSR1, and MARCO. Notably, KCR pretreatment attenuated CSF2‐induced JAK2/STAT5 phosphorylation, ROS production, and phagocytosis, consistent with competitive interference with endogenous CSF2‐CSF2R signaling. Together, these findings reveal KCR as a previously unrecognized *T. gondii* factor that targets the CSF2/CSF2R axis to recalibrate macrophage effector functions, expanding the repertoire of parasite strategies for cytokine pathway modulation and highlighting CSF2‐CSF2R signaling as a potential interface for mechanistic and therapeutic investigation in toxoplasmosis.

## 1. Introduction

Toxoplasmosis, caused by the apicomplexan protozoan *Toxoplasma gondii*, is among the most prevalent zoonotic infections worldwide. The parasite infects a wide range of warm‐blooded hosts and is associated with adverse outcomes such as miscarriage, stillbirth, congenital abnormalities, and neurological sequelae. Epidemiological estimates suggest that approximately one‐third of the global population has been exposed to or infected with *T. gondii* [1, 2]. In livestock systems, toxoplasmosis imposes substantial economic burdens through direct losses from morbidity and mortality. Additionally, infection during pregnancy leads to indirect losses driven by reproductive failure, including abortion, stillbirth, and diminished offspring viability [3, 4].

The host immune response to *T. gondii* infection is mediated by a complex cytokine network. Following invasion, tachyzoites elicit a robust T helper 1 (Th1)‐biased response characterized by the production of interferon (IFN)‐γ, interleukin (IL)‐12, and tumor necrosis factor (TNF)‐α, which are essential for controlling acute infection and eliminating most invading parasites [2]. Extensive in vitro and in vivo evidence underscores the central role of cytokine‐mediated immunity in limiting toxoplasmosis. For instance, IFN‑γ induces immunity‐related GTPases (IRGs) [5] and guanylate‑binding proteins (GBPs) [6], promoting disruption of the parasitophorous vacuole (PV) and restricting parasite survival [7]. IL‑12 acts as an early pro‑inflammatory signal that initiates and shapes the adaptive immune response against *T. gondii* [8]. Produced primarily by dendritic cells (DCs) and macrophages, IL‑12 supports the expansion and cytotoxic function of natural killer (NK) cells, as well as CD4^+^ and CD8^+^ T cells, and it drives robust IFN‑γ production [9]. Moreover, *T. gondii* profilin‑like protein engages murine Toll‐like receptor (TLR) 11, leading to DC activation and a strong MyD88‑dependent IL‑12 response [10]. Despite these defenses, *T. gondii* has evolved sophisticated immune‐evasion strategies. Virulence effectors secreted from rhoptries (ROPs) [11–13] and dense granules (GRAs) [14–16] can modulate host IFN‐γ, IL‐12 and TNF‐α responses, thereby facilitating intracellular survival, replication, and disease pathogenesis.

The production of host cytokine mimics constitutes an additional strategy by which protozoan parasites modulate host cytokine signaling. Several protozoa, including *Plasmodium* [17], *Leishmania* [18], *Entamoeba histolytica* [19, 20], and *T. gondii* [21], have been reported to encode macrophage migration inhibitory factor (MIF) homologs that are structurally similar to human MIF. These parasite‐derived MIF proteins can engage the human MIF receptor (CD74), activate downstream ERK1/2 and PI3K/Akt signaling, and regulate the secretion of cytokines such as TNF‐α, IL‐8, and IL‐12 by immune and epithelial cells, thereby facilitating immune evasion, tissue invasion, and disease pathogenesis [22]. Collectively, these observations support the notion that deployment of effector proteins to reshape the host inflammatory and immune microenvironment is a conserved feature of host‐*T. gondii* interactions.

Given its global prevalence and obligate intracellular lifestyle, it is pertinent to ask whether *T. gondii* encodes additional proteins capable of modulating key host cytokine responses beyond the well‐characterized TgMIF, ROPs, and GRAs. Defining such effector repertoires is essential for elucidating the mechanisms by which *T. gondii* broadly reprograms host immune responses. Granulocyte‐macrophage colony‐stimulating factor (GM‐CSF/CSF2) is a pleiotropic cytokine that modulates the proliferation, differentiation, and activation of myeloid cells, including macrophages and neutrophils. CSF2 signaling is initiated by engagement of its receptor (CSF2R), which activates the JAK‐STAT pathway [23, 24]. Downstream signaling promotes the production of pro‐inflammatory mediators such as IL‐6 and TNF‐α, thereby amplifying inflammatory cell activation and expansion via positive feedback mechanisms [25, 26]. In addition, JAK‐STAT activation enhances NADPH oxidase activity, increasing reactive oxygen species (ROS) generation and contributing to antimicrobial effector functions [27]. Notably, host CSF2 alone, in the absence of IL‐12p70 or IFN‐γ stimulation, can restrict *T. gondii* replication in murine microglia by promoting ROS and nitric oxide (NO) production [28]. In the present study, we identified a *T. gondii*‐encoded 3‐ketoacyl‐CoA reductase (KCR; ToxoDB: TGME49_271888) that interacted with CSF2Rα, thereby modulating CSF2/CSF2R axis‐mediated JAK2/STAT5 signaling as well as JAK2/STAT5‐dependent ROS production and phagocytic activity. Given the pivotal roles of ROS and phagocytosis in parasite killing and clearance [29], KCR may facilitate *T. gondii* infection and support latent persistence.

## 2. Materials and Methods

### 2.1. Animals, Cells, and Parasites

Specific pathogen‐free BALB/c mice (20–25 g) were obtained from Henan Skobes Biotechnology Co., Ltd. (Anyang, Henan, China) and housed in the Experimental Animal Center of Nanjing Agricultural University (Protocol number: NJAU.No20230911131). Human foreskin fibroblasts (HFFs), HEK 293T cells, and murine macrophage RAW264.7 cells were purchased from the American Type Culture Collection and are currently maintained in our laboratory. Tachyzoites of the *T. gondii* RH strain were cryopreserved in cell‐freezing medium under liquid nitrogen and routinely propagated by serial passage in HFFs, with virulence maintained by periodic intraperitoneal passage in BALB/c mice.

### 2.2. Antibodies

Flag‐tag mouse monoclonal antibody (mAb) (#M20008), His‐tag mouse mAb (#M20001), and mouse IgG (#B30010M) were purchased from Abmart Biotech, Inc. (Shanghai, China). Flag‐tag rabbit mAb (#20543‐1‐AP), His‐tag rabbit pAb (#10001‐0‐AP), and beta actin mouse mAb (#66009‐1‐Ig) were purchased from Proteintech Biotech, Inc. (Wuhan, Hubei, China). Goat Anti‐Mouse IgG (H + L) HRP (#BS12478) and Goat Anti‐Rabbit IgG (H + L) HRP (#BS13278) were purchased from Bioworld Biotech, Inc. (Nanjing, Jiangsu, China). Jak2 (D2E12) Rabbit mAb (#4040), Stat5 (D2O6Y) Rabbit mAb (#94205), and Phospho‐Stat5 (Tyr694) (C11C5) Rabbit mAb (#9359) were purchased from Cell Signaling Technology (Boston, MA, USA). Phospho‐JAK2 (Y1007) pAb (#YP0155) was purchased from Immunoway Biotech, Inc. (San Jose, CA, USA). Anti‐Mouse CSF2 Antibody (MP1‐22E9) (#GC27003) was purchased from GlpBio Biotech, Inc. (Montclair, CA, USA).

### 2.3. In Vitro Cultivation of *T. gondii* RH Tachyzoites

HFF cells were used for in vitro propagation of *T. gondii* RH strain tachyzoites. Cells were cultured in complete Dulbecco’s modified Eagle’s medium (DMEM; Gibco, Waltham, MA, USA) supplemented with 10% fetal bovine serum (FBS; Cytiva, Marlborough, MA, USA) and 1% penicillin–streptomycin (Gibco, Thermo Fisher Scientific, Waltham, MA, USA). At ~80% confluence, 5 × 10^5^ tachyzoites were inoculated into T25 cell culture flasks. When parasite egress was evident, the monolayer was dislodged using a cell scraper, and the suspension was sequentially passed through 5‐mL and 1‐mL syringe needles to mechanically disrupt host cells. The resulting lysate was then filtered through 5‐µm membrane filters to remove host cells and large debris, yielding a highly enriched tachyzoite preparation.

### 2.4. Eukaryotic Plasmid Construction and Protein Expression

Total RNA was extracted from RH strain tachyzoites and murine splenocytes, respectively, using RNA Isolater Total RNA Extraction Reagent (Vazyme, Nanjing, China) and reverse‐transcribed into cDNA using HiScript IV 1st Strand cDNA Synthesis Kit (+gDNA wiper) (Vazyme, Nanjing, China). The resulting cDNA was used as the template to amplify the *T. gondii KCR* gene (TGME49_271888) and the murine *CSF2Rα* gene (Accession Number NM_009970.2), respectively, using the primers listed in Supporting Information 1: Table S1. PCR products (Supporting Information 2) were cloned into the pcDNA3.1 and pCAGGS plasmids by homologous recombination to generate pcDNA3.1‐KCR (Flag‐tagged) or pCAGGS‐CSF2Rα (His‐tagged). The resulting recombinant plasmids were transformed into *Escherichia coli* DH5α competent cells, and positive clones were identified by colony PCR and confirmed by nucleotide sequencing. Sequence identities were further verified by BLAST analysis (https://toxodb.org/toxo/app/workspace/blast; http://www.ncbi.nlm.nih.gov/BLAST) against ToxoDB and NCBI databases.

The recombinant plasmids were transfected into HEK 293T cells using Lipofectamine 2000 (Invitrogen, Carlsbad, CA, USA) to verify protein expression. At 24 h posttransfection, cells were lysed with NP‐40 lysis buffer (Thermo Fisher Scientific, Waltham, MA, USA) supplemented with a protease inhibitor cocktail (Beyotime Biotechnology, Shanghai, China). Lysates were clarified by high‐speed centrifugation at 4°C, and supernatants were subjected to SDS‐PAGE followed by transfer to PVDF membranes (Merck, Darmstadt, Hessen, Germany). Immunoblotting was performed with anti‐Flag (1:5000) or anti‐His mouse mAbs (1:5000), and signals were detected using Chemistar High‐sig ECL Substrate (Tannon, Shanghai, China) to assess eukaryotic protein expression. Flag‐tagged *T. gondii* KCR protein was subsequently purified using Anti‐DYKDDDDK Magarose Beads (Smart‐Lifesciences, Changzhou, China) according to the manufacturer’s instructions.

### 2.5. Co‐Immunoprecipitation (Co‐IP) Analysis of the Interaction Between KCR and Murine CSF2Rα

HEK 293T cells were co‐transfected with 2 μg each of recombinant eukaryotic plasmids encoding KCR and murine CSF2Rα. After transfection, cells were lysed in NP‐40 lysis buffer, and lysate was clarified by centrifugation. The resulting supernatants were divided into four aliquots: one aliquot served as the input control to verify protein expression, and the remaining three aliquots were subjected to Co‐IP assays, including a forward Co‐IP (anti‐KCR), a reverse Co‐IP (anti‐CSF2Rα), and a normal mouse IgG control to assess nonspecific binding. For the forward Co‐IP, supernatants were incubated with anti‐Flag mouse mAbs to immunoprecipitate KCR, whereas for the reverse Co‐IP, supernatants were incubated with anti‐His mouse mAbs to immunoprecipitate murine CSF2Rα. For the IgG control, supernatants were incubated with normal mouse IgG.

In each Co‐IP condition, antibody‐lysate mixtures were incubated with continuous rotation at 4°C for 12 h, followed by addition of 20 µL Protein A/G magnetic beads (Vazyme, Nanjing, China) and a further 4‐h incubation at 4°C with rotation to capture immune complexes. Beads were washed three times and resuspended in PBS. The bead‐bound complexes were then divided into two equal fractions for immunoblotting: one fraction was probed with anti‐Flag rabbit mAbs (1:3000) and the other with anti‐His rabbit pAbs (1:1000) to evaluate the interaction between KCR and murine CSF2Rα.

### 2.6. Modulation of the JAK2/STAT5 Pathway by KCR in RAW264.7 Macrophages

To determine whether KCR recapitulates CSF2 bioactivity, we examined its ability to activate CSF2/CSF2R‐dependent JAK2/STAT5 signaling in RAW264.7 macrophages. RAW264.7 cells were seeded in 6‐well plates and treated with either KCR protein or murine CSF2 (#ab259385; Abcam, Cambridge, Cambridgeshire, UK) at a final concentration of 200 ng/mL for 0, 15, and 30 min at 37°C with 5% CO_2_. Each condition was performed in triplicate. Following stimulation, cells were washed with ice‐cold PBS and lysed in RIPA Lysis and Extraction Buffer (Thermo Fisher Scientific, Waltham, MA, USA) supplemented with protease and phosphatase inhibitors (NCM Biotech, Suzhou, China). Total cellular proteins were extracted, and 30 µg of protein per sample was subjected to western blot analysis. Primary antibodies against phosphorylated JAK2 (p‐JAK2, 1:500), total JAK2 (1:1000), p‐STAT5 (1:1000), total STAT5 (1:1000), and β‐actin (loading control, 1:30,000) were used. Signals were developed using Chemistar High‐sig ECL Western Blotting Substrate, and band intensities were quantified using ImageJ software (National Institutes of Health, Bethesda, MD, USA). Fold changes in p‐JAK2 and p‐STAT5 levels relative to baseline (0 min) were calculated for each time point following KCR or murine CSF2 stimulation.

### 2.7. Measurement of Intracellular ROS Levels

RAW264.7 cells were seeded in 96‐well plates and treated with either recombinant KCR protein or murine CSF2 at final concentrations of 0, 50, 100, and 200 ng/mL for 8 h. Lipopolysaccharide (LPS; Sigma–Aldrich, St. Louis, MO, USA) at 500 ng/mL was included as a positive control. All conditions were performed in triplicate. Intracellular ROS levels were quantified using the fluorescent probe DCFH‐DA according to the manufacturer’s instructions (Beyotime Biotechnology, Nanjing, China). Fluorescence intensity was determined using a multifunction microplate reader with excitation/emission wavelengths of 488 and 525 nm, respectively.

### 2.8. Analysis of mRNA Expression of NADPH Oxidase Subunit Gene

RAW264.7 cells were seeded in 6‐well plates and stimulated with either recombinant KCR protein or murine CSF2 at a final concentration of 200 ng/mL for 6 h. PBS‐treated cells served as the negative control. Each condition was performed in triplicate. Total RNA was extracted using RNA Isolater Total RNA Extraction Reagent (Vazyme) and reverse‐transcribed into cDNA from 1 μg RNA using the HiScript IV RT SuperMix for qPCR (+gDNA wiper) (Vazyme) following the manufacturer’s protocol. Quantitative PCR (qPCR) was performed using ChamQ Blue Universal SYBR qPCR Master Mix (Vazyme) with prepared cDNA as templates according to the manufacturer’s instructions. The transcriptional levels of NADPH oxidase subunit genes CYBB, CYBA, NCF1, and NCF2 were evaluated using the 2^-ΔΔCt^ method. All primers used for qPCR are listed in Supporting Information 1: Table S2.

### 2.9. Measurement of Phagocytic Capacity by FITC‐Dextran Uptake

RAW264.7 cells were seeded in 24‐well plates and treated for 12 h with either recombinant KCR protein or murine CSF2 at a final concentration of 200 ng/mL. LPS (500 ng/mL) stimuli were included as a positive control, and all conditions were performed in triplicate. Following stimulation, culture medium was removed and replaced with 250 µL of fresh medium supplemented with 250 µL of FITC‐dextran (GC19939; 1 mg/mL, GlpBio Biotech). Cells were incubated for 1 h at 37°C in the dark. Subsequently, cells were washed three times with PBS to remove any noninternalized FITC‐dextran and analyzed by flow cytometry. The phagocytic capacity was quantified as the mean fluorescence intensity (MFI) of FITC‐positive cells, with higher MFI values indicating increased uptake. The phagocytosis index was calculated as the ratio of the MFI of each treatment group to that of the mock control.

### 2.10. Analysis of mRNA Expression of Phagocytic Receptor Gene

RAW264.7 cells seeded in 6‐well plates were treated for 6 h with PBS (mock control), recombinant murine CSF2, or KCR protein, with each treatment performed in triplicate. Cells were harvested, and total RNA was extracted and reverse‐transcribed into cDNA. The qPCR assay was subsequently performed to assess the transcriptional levels of genes encoding phagocytic receptors, including the complement receptor CR3, the Fc receptors CD16 and CD64, and the scavenger receptors MSR1 and MARCO. Primer sequences are provided in Supporting Information 1: Table S2.

### 2.11. Evaluation of KCR‐Mediated Antagonism of CSF2‐Induced JAK2/STAT5 Activation, ROS Production, and Phagocytosis

To assess whether KCR competitively inhibits murine CSF2‐CSF2R signaling, RAW264.7 cells were assigned to five groups (*n* = 3 independent replicates per group): (1) mock control, (2) KCR alone, (3) KCR plus CSF2, (4) CSF2 alone, and (5) CSF2 plus CSF2 inhibitor (anti‐mouse CSF2 antibody). Cells in the KCR plus CSF2 group were preincubated with KCR for 1 h to allow the binding of KCR to CSF2R and thereby limit subsequent CSF2‐CSF2R engagement. Cells were then incubated with a panel of treatments, including PBS (group 1), KCR (200 ng/mL; group 2), CSF2 (200 ng/mL; groups 3 and 4), or CSF2 (200 ng/mL) together with the CSF2 inhibitor (8 μg/mL; group 5). Incubation times were assay‐dependent: 15 min for immunoblotting, 8 h for ROS quantification, and 12 h for phagocytosis analysis. For analysis of JAK2/STAT5 phosphorylation, cells were harvested after 15 min, lysed in RIPA buffer, and total protein was extracted. Equal amounts of protein (30 μg) were resolved by SDS‐PAGE and transferred to PVDF membranes. Membranes were probed with antibodies against phosphorylated JAK2 (Tyr1007) and STAT5 (Tyr694), along with corresponding total JAK2 and STAT5 antibodies, and β‐actin served as the loading control. Signals were developed using a Chemistar High‐sig ECL substrate, and band intensities were quantified by grayscale analysis with ImageJ to determine whether KCR pretreatment antagonizes CSF2‐induced JAK2/STAT5 activation. Intracellular ROS levels were measured after 8 h using the DCFH‐DA fluorescent probe according to the manufacturer’s instructions, and fluorescence was quantified using a multifunction microplate reader to evaluate whether KCR competitively reduces CSF2‐induced ROS production. Phagocytic capacity was assessed after 12 h of treatment. Cells were incubated with FITC‐dextran and analyzed by flow cytometry and the MFI of FITC was used to quantify phagocytosis and to determine whether KCR pretreatment inhibits CSF2‐stimulated uptake.

### 2.12. Statistical Analysis of Data

Statistical analyses were performed using GraphPad Prism 10.6.0 (GraphPad software, San Diego, CA, USA). Differences among treatment groups were assessed by ordinary one‐way ANOVA followed by Dunnett’s multiple‐comparison test. Data are presented as mean ± standard deviation (SD). Statistical significance was defined as  ^∗^ *p* < 0.05,  ^∗∗^ *p* < 0.01,  ^∗∗∗^ *p* < 0.001, and  ^∗∗∗∗^ *p* < 0.0001.

## 3. Results

### 3.1. Identification of *T. gondii* KCR and Co‐IP Assays Confirmed Its Interaction With Murine CSF2Rα

**Figure 1:**
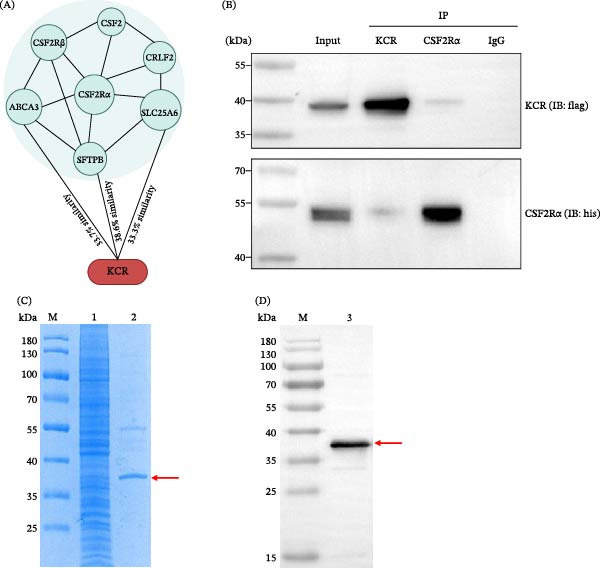
Protein interactions and eukaryotic protein acquisition. (A) Schematic overview of the screening strategy and identification of *T. gondii* KCR. (B) Co‐immunoprecipitation identification of the interaction between KCR and murine CSF2Rα input: cell lysates from HEK 293T cells co‐transfected with pcDNA3.1‐KCR and pCAGGS‐CSF2R for 24 h; IP: KCR, CSF2α or IgG: immunoprecipitation was performed using Flag‐tag mouse mAb, His‐tag mouse mAb or mouse IgG; IB: KCR or CSF2α: immunoblot analysis was performed using Flag‐tag rabbit mAb or His‐tag rabbit pAb. (C) Acquisition of KCR eukaryotic protein. Lane M: standard molecular marker for protein; lane 1: cell lysates from HEK 293T cells transfected with pcDNA3.1‐KCR for 24 h; lane 2: purified KCR eukaryotic protein. (D) Western blot analysis of KCR M: standard molecular marker for protein; lane 3: his‐tag in purified KCR was identified by His‐tag mouse mAb.

To identify candidate *T. gondii* effector proteins that may modulate CSF2‐CSF2R signaling, we queried ToxoDB using the amino acid sequences of SFTPB, ABCA3, and SLC25A6, which are predicted by STRING (https://cn.string-db.org/) to interact with CSF2Rα. This screening identified the *T. gondii* KCR, which exhibits moderate sequence similarity to SFTPB, ABCA3, and SLC25A6 (Figure 1A). To investigate the interaction between KCR protein and murine CSF2Rα, recombinant pcDNA3.1‐KCR and pCAGGS‐CSF2Rα vectors were co‐transfected into HEK 293T cells. Cell lysates were subjected to forward and reverse Co‐IP assays, and protein–protein interactions were evaluated by western blotting. In the input samples, both KCR and murine CSF2Rα were readily detected using anti‐Flag‐tag rabbit mAbs and anti‐His‐tag rabbit pAbs (Figure 1B), respectively, confirming robust expression of both KCR and CSF2Rα proteins following co‐transfection. In the forward Co‐IP using anti‐Flag‐tag mouse mAbs, KCR was successfully precipitated and detected with anti‐Flag‐tag rabbit mAb, and the co‐precipitated CSF2Rα was detected with anti‐His‐tag rabbit pAbs (Figure 1B). Conversely, in the reverse Co‐IP using anti‐His‐tag mouse mAbs, CSF2Rα was precipitated, and the associated KCR was detected with anti‐Flag‐tag rabbit mAbs (Figure 1B). In the control immunoprecipitations performed with normal mouse IgG, neither anti‐Flag‐tag rabbit mAbs nor anti‐His‐tag rabbit pAbs detected KCR or CSF2Rα signals (Figure 1B). Collectively, these results demonstrate a specific interaction between the *T. gondii* KCR and murine CSF2Rα. Although the co‐precipitated signal intensity was modest, the interaction was reproducibly detected in independent experiments, supporting the possibility that KCR may engage the CSF2R pathway in a CSF2‐like manner.

### 3.2. Eukaryotic Expression, Purification, and Western Blot Validation of KCR

To further characterize KCR and facilitate functional analyses as a putative mimic of murine CSF2, we transfected recombinant pcDNA3.1‐KCR plasmids into HEK 293T cells for eukaryotic expression and subsequent purification. The SDS‐PAGE results showed that compared to the crude cell lysate (Figure 1C, lane 1), the purified KCR protein migrated as a distinct single band at ~ 37 kDa (Figure 1C, lane 2), consistent with the predicted molecular mass. Western blotting further verified the identity of the purified protein, which was specifically recognized by anti‐His‐tag mouse mAbs (Figure 1D, lane 3) and exhibited the same apparent molecular weight (~37 kDa) as observed by SDS‐PAGE.

### 3.3. KCR Acts as a CSF2 Mimic to Activate JAK2/STAT5 Signaling

**Figure 2:**
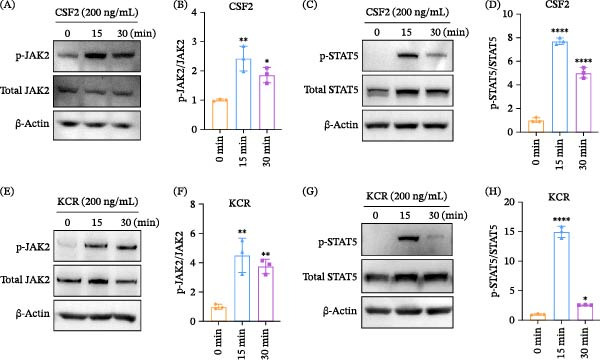
Activation of the JAK2/STAT5 pathway by KCR in macrophages. (A, C) Western blot analysis of phosphorylated JAK2 (p‐JAK2) and STAT5 (p‐STAT5) in RAW264.7 mouse macrophages stimulated with murine CSF2. (B, D) Densitometric quantification of p‐JAK2 and p‐STAT5 levels from (A) and (C), respectively. (E, G) Western blot analysis of p‐JAK2 and p‐STAT5 in RAW264.7 macrophages stimulated with KCR. (F, H) Densitometric quantification of p‐JAK2 and p‐STAT5 levels from (E) and (G), respectively.  ^∗^ *p* < 0.05,  ^∗∗^ *p* < 0.01, and  ^∗∗∗∗^ *p* < 0.0001.

Given that the binding of CSF2 to its receptor CSF2R triggers JAK2/STAT5 signaling, we investigated whether KCR could elicit a similar response. Phosphorylation levels of the key signaling pathway components JAK2 and STAT5 were quantified by western blotting. As expected, murine CSF2 stimulation significantly increased JAK2 and STAT5 phosphorylation in RAW264.7 macrophages, with a peak response at 15 min (*p* < 0.01) (Figure 2A–D). KCR elicited a comparable phosphorylation profile, significantly enhancing JAK2 and STAT5 phosphorylation within 15–30 min (*p* < 0.01) and exhibiting maximal activation at 15 min (Figure 2E–H). Collectively, these data suggested that KCR engagement of murine CSF2Rα was sufficient to activate the canonical JAK2/STAT5 signaling cascade.

### 3.4. KCR Promotes NADPH Oxidase‐Dependent ROS Production

**Figure 3:**
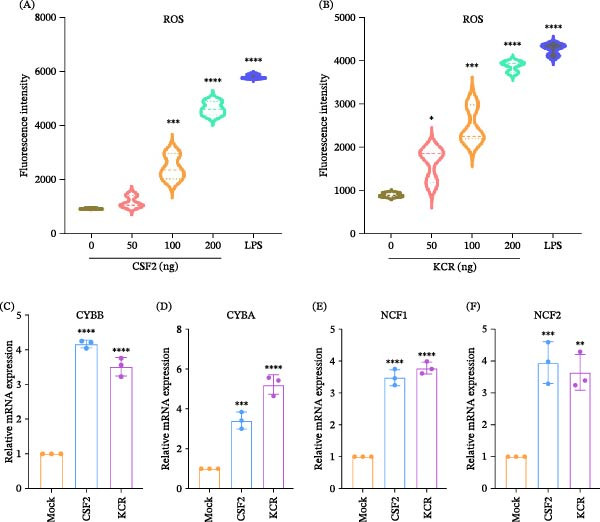
Enhancement of the ROS production based on NADPH oxidase catalysis by KCR. (A) ROS production in murine macrophages treated with CSF2. (B) ROS production in murine macrophages treated with KCR. (C–F) Transcription levels of the NADPH oxidase subunits gene CYBB, CYBA, NCF1, and NCF2 in murine macrophages following KCR stimulation.  ^∗^ *p* < 0.05,  ^∗∗^ *p* < 0.01,  ^∗∗∗^ *p* < 0.001, and  ^∗∗∗∗^ *p* < 0.0001.

To determine whether KCR‐induced activation of the JAK2/STAT5 pathway is associated with NADPH oxidase‐mediated ROS generation, intracellular ROS levels and NADPH oxidase subunit gene expression were assessed by fluorescence‐based ROS assays and qPCR, respectively. As a positive control, 500 ng/mL LPS significantly increased ROS production (Figure 3A,B, *p* < 0.0001). KCR, similar to murine CSF2, significantly enhanced ROS generation at 50, 100, and 200 ng/mL (Figure 3A,B, *p* < 0.05). As for the core NADPH oxidase subunits implicated in oxidative burst responses, CSF2 stimulation significantly increased the transcript levels of CYBB (*p* < 0.0001; Figure 3C), CYBA (*p* < 0.001; Figure 3D), NCF1 (*p* < 0.0001; Figure 3E), and NCF2 (*p* < 0.001; Figure 3F). Consistently, KCR treatment also upregulated CYBB (*p* < 0.0001; Figure 3C), CYBA (*p* < 0.0001; Figure 3D), NCF1 (*p* < 0.0001; Figure 3E), and NCF2 (*p* < 0.01; Figure 3F), to levels comparable to those induced by CSF2. Collectively, these data indicate that KCR‐CSF2Rα engagement activates JAK2/STAT5 signaling and promotes NADPH oxidase‐dependent ROS production.

### 3.5. KCR Enhances Complement/Fc/Scavenger Receptor‐Mediated Phagocytosis

**Figure 4:**
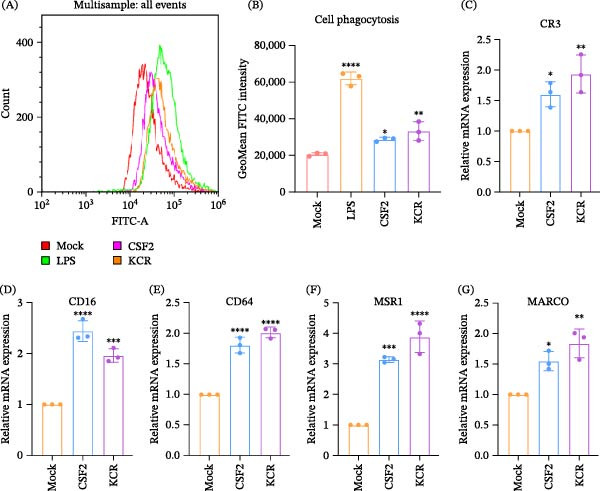
Promotion of phagocytosis mediated by complement/Fc/scavenger receptor by KCR. (A) Enhancement of mean FITC intensity in contour plot of flow cytometry by KCR. (B) Phagocytosis in murine macrophages stimulated with CSF2. (C–G) Transcription levels of complement (CR3), Fc (CD16 and CD64), and scavenger receptor (MSR1 and MARCO) in murine macrophages following KCR stimulation.  ^∗^ *p* < 0.05,  ^∗∗^ *p* < 0.01,  ^∗∗∗^ *p* < 0.001, and  ^∗∗∗∗^ *p* < 0.0001.

To determine whether KCR‐driven activation of the JAK2/STAT5 pathway augments complement/Fc/scavenger receptor‐dependent phagocytosis, FITC‐dextran uptake was quantified by flow cytometry, and receptor gene expression was assessed by qPCR. As shown in Figure 4A,B, LPS stimulation (500 ng/mL) markedly increased FITC‐dextran uptake by RAW264.7 macrophages (*p* < 0.0001), and murine CSF2 (200 ng/mL) similarly enhanced uptake (*p* < 0.05). Notably, KCR (200 ng/mL) also significantly increased FITC‐dextran internalization (Figure 4A,B), indicating an augmented phagocytic capacity (*p* < 0.01). In parallel, CSF2 treatment significantly upregulated transcripts encoding phagocytic receptors, including CR3 (*p* < 0.05; Figure 4C), CD16 (*p* < 0.0001; Figure 4D), CD64 (*p* < 0.0001; Figure 4E), MSR1 (*p* < 0.001; Figure 4F), and MARCO (*p* < 0.05; Figure 4G). KCR elicited a comparable transcriptional response, significantly increasing CR3 (*p* < 0.01; Figure 4C), CD16 (*p* < 0.001; Figure 4D), CD64 (*p* < 0.0001; Figure 4E), MSR1 (*p* < 0.0001; Figure 4F), and MARCO (*p* < 0.01; Figure 4G). Collectively, these data suggested that KCR‐CSF2Rα engagement activated JAK2/STAT5 signaling and potentiated phagocytosis mediated by complement, Fc, and scavenger receptors.

### 3.6. KCR Competitively Antagonized CSF2‐Induced JAK2/STAT5 Activation, ROS Generation and Phagocytosis

**Figure 5:**
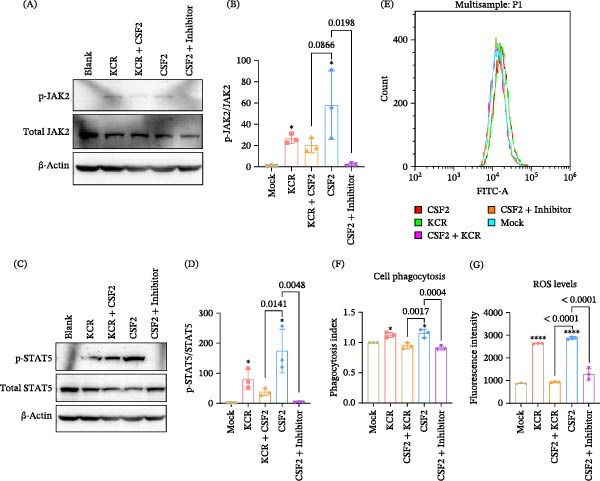
Competitive attenuation of murine CSF2‑induced JAK2/STAT5 activation, ROS production, and phagocytosis by KCR. (A, C) Western blot analysis of competitive attenuation of murine CSF2‐induced p‐JAK2 and p‐STAT5 in macrophages by KCR. (B, D) Densitometric quantification of p‐JAK2 and p‐STAT5 levels from (A) and (C), respectively. (E) Competitive attenuation of CSF2‐induced mean FITC intensity in contour plot of flow cytometry by KCR. (F) Competitive attenuation of CSF2‐induced phagocytosis in macrophages by KCR. (G) Competitive attenuation of CSF2‐induced ROS production in macrophages by KCR.  ^∗^ *p* < 0.05 and  ^∗∗∗∗^ *p* < 0.0001.

We pretreated RAW264.7 cells with KCR to assess whether prior receptor engagement by KCR could interfere with subsequent CSF2‐driven signaling and then re‐evaluated CSF2‐driven JAK2/STAT5 activation, ROS production, and phagocytosis. With respect to JAK2/STAT5 signaling, pretreatment with the CSF2‐specific inhibitor (anti‐CSF2 antibody) nearly abolished CSF2‑induced phosphorylation of JAK2 (Figure 5A,B, *p* = 0.0198) and STAT5 (Figure 5C,D, *p* = 0.0048). Pretreatment with KCR also reduced CSF2‑triggered phosphorylation of JAK2 (Figure 5A,B, *p* = 0.0866) and STAT5 (Figure 5C,D, *p* = 0.0141), albeit to a lesser extent than the CSF2 neutralizing antibody. In parallel, phagocytosis induced by CSF2 was also markedly suppressed by pretreatment with KCR (Figure 5E,F, *p* = 0.0017) or the CSF2‐specific inhibitor (Figure 5E,F, *p* = 0.0004). Consistently, both KCR (*p* < 0.0001) and the CSF2‐specific inhibitor (*p* < 0.0001) significantly reduced CSF2‑stimulated ROS generation (Figure 5G). Collectively, these findings supported a competitive interaction in which KCR interfered with CSF2 binding to CSF2Rα, thereby dampening downstream signaling and effector functions, including JAK2/STAT5 activation, ROS production, and phagocytosis. Although KCR pretreatment reduced CSF2‐induced JAK2 phosphorylation, this reduction did not reach statistical significance under the current sample size. This likely reflects the transient kinetics and inherent variability of proximal kinase activation relative to downstream STAT5 signaling, as well as limited statistical power. Importantly, downstream STAT5 activation and functional outputs were significantly attenuated, indicating biologically meaningful modulation at the receptor signaling level.

## 4. Discussion

In this study, we uncovered a previously unappreciated immunomodulatory activity of *T. gondii* KCR. Through co‑immunoprecipitation assays, KCR was shown to interact with host CSF2Rα, indicating that KCR can engage the CSF2R receptor axis. Functionally, exogenous KCR stimulated rapid phosphorylation of JAK2 and STAT5 in murine macrophages while enhancing both NADPH oxidase‑mediated ROS production and phagocytic receptor‑mediated phagocytic capacity. Notably, KCR pretreatment attenuated murine CSF2‑induced activation of JAK2/STAT5, ROS generation, and phagocytosis, supporting a competitive relationship between KCR and host CSF2 for binding to CSF2R. Collectively, these findings extend the repertoire of *T. gondii* effectors known to manipulate host cytokine signaling beyond the well‑characterized TgMIF family and implicate the CSF2/CSF2R axis as a previously unrecognized target exploited by the parasite to dampen host immune responses and facilitate immune evasion.

Accumulating evidence supports the biological plausibility that parasite‐derived factors may target CSF2R‐dependent pathways. *T. gondii* infection triggers the production of CSF2 in various host tissues and targets the CSF2/CSF2R axis, contributing to a local inflammatory environment and shaping the proliferation, differentiation, and activation states of myeloid cells. For example, CSF2 secreted by *T. gondii*‐infected human fibroblasts acts as a potent stimulus that delays neutrophil apoptosis for up to 72 h. This enhanced neutrophil survival may contribute to the robust proinflammatory response elicited in the *T. gondii*‐infected host [30]. Stimulation of *T. gondii*‐infected murine cerebellar neurons with exogenous IFN‑γ and/or TNF failed to suppress parasite invasion and replication, yet these neurons continued to produce CSF2 [31]. This supports the view that CSF2 driven by *T. gondii* infection may originate from multiple cells beyond classical immune cells. Second, CSF2 is associated with enhanced antiparasitic capability in myeloid cells. In vitro, CSF2 alone activates murine microglia, which is associated with subsequent reduced parasite replication. It is accompanied by the production of TNF‐α and IL‐6, as well as NO and ROS effects, even in the absence of IL‐12p70 or IFN‐γ stimulation [28]. Conceptually, these observations support a host defense mechanism in which CSF2 polarizes macrophages and microglia toward a more inflammatory and antipathogen active state, primarily manifested by the secretion of IFN‑γ, enhances phagocytosis, and promotes the release of ROS and NO [32–35]. In this context, we identified KCR as a candidate CSF2 modulator that interacts with murine CSF2Rα, supported by bioinformatics analyses and Co‐IP validation. These results suggest that *T. gondii* may engage CSF2Rα during infection and thereby activate and/or recalibrate CSF2/CSF2R signaling to modulate host innate immune responses. To be noted, the KCR‐CSF2Rα interaction was demonstrated in an overexpression system and could be substantiated by quantitative binding approaches that assess affinity and specificity for future investigation.

Parasite‐encoded cytokine mimics, such as the MIF homologs reported in various protozoan parasites, have been proposed to reshape host inflammatory circuits by directly targeting host cytokine receptors and downstream signaling pathways to facilitate immune evasion, invasion, and pathogenesis [21, 22]. In this study, we advance the concept that KCR functions as a noncanonical cytokine mimic. Although annotated as a metabolic enzyme and sharing only moderate amino acid homology with mouse CSF2 (data not shown), KCR exhibits ligand‐like activity consistent with receptor engagement. Such molecular mimicry may be advantageous for *T. gondii*, an obligate intracellular parasite that must finely tune myeloid immunity to balance replication, dissemination, and persistence. Mechanistically, our data directly support this by demonstrating that KCR triggers a CSF2Rα–JAK2/STAT5 signaling cascade in macrophages. CSF2R, composed of α and β subunits, is activated upon CSF2 binding, triggering key signaling pathways, including JAK/STAT, PI3K/Akt, and ERK1/2 MAPK, that are critical for immune activation [36, 37]. CSF2 has been reported to enhance phagocytosis, ROS production, and CD11b expression [38, 39], with JAK2 and STAT5 serving as key effectors of the canonical JAK/STAT arm. Consistent with the confirmed interaction between KCR and host CSF2Rα, KCR, similar to CSF2, induced rapid phosphorylation of JAK2 and STAT5 in RAW264.7 macrophages within 30 min. Downstream functional readouts further corroborated CSF2‐like activity. KCR increased intracellular ROS levels in a dose‐dependent manner and significantly upregulated transcripts encoding core NADPH oxidase components (CYBB, CYBA, NCF1, and NCF2), consistent with activation of an oxidative burst program. KCR also enhanced FITC‐dextran uptake by macrophages, indicating increased phagocytic capacity. Importantly, KCR specifically elevated expression of genes encoding complement (CR3), Fc (CD16 and CD64), and scavenger receptors (MSR1 and MARCO), suggesting that augmented phagocytosis reflects coordinated upregulation of multiple receptor systems rather than nonspecific activation [40]. Together, these findings indicate that KCR can modulate host CSF2 signaling by engaging CSF2Rα, thereby influencing downstream antimicrobial effector responses. However, the physiological mode by which KCR is delivered to host receptors, whether through parasite lysis, nonclassical secretion, extracellular vesicles, or exposure at the host–parasite interface, requires further clarification to establish its role as a bona fide effector.

From an alternative perspective, KCR targets CSF2Rα, a receptor subunit whose engagement by CSF2 is crucial for macrophage development and function [41]. This observation raises the possibility that, during *T. gondii* infection, KCR competes with endogenous CSF2 for CSF2Rα binding, potentially generating complex immunomodulatory consequences. On the one hand, such competition could blunt optimal CSF2‐driven immune activation and thereby favor parasite immune evasion. On the other hand, the partial agonistic activity of KCR on JAK2/STAT5 signaling may sustain a tempered inflammatory state that supports parasite persistence. Consistent with this model, KCR pretreatment significantly reduced, yet did not completely abrogate, CSF2‐induced JAK2/STAT5 phosphorylation, ROS production, and phagocytic activity. Mechanistically, several nonmutually exclusive models may explain the attenuation of CSF2‐induced signaling following KCR pretreatment. First, KCR may directly compete with CSF2 for binding to CSF2Rα, thereby limiting ligand access. Second, KCR engagement may induce a partially active or biased receptor conformation that alters downstream signaling output. Third, KCR binding to CSF2Rα may interfere with proper assembly of the CSF2Rα‐CSF2Rβ signaling complex, which is required for optimal JAK2 activation. Fourth, KCR may promote receptor desensitization or internalization, reducing subsequent responsiveness to CSF2. Future quantitative binding studies and receptor trafficking analyses are needed to distinguish among these possibilities. To be noted, these findings are conceptually aligned with precedents from viral immune modulation. In HIV‐1 infection, viral proteins have been reported to suppress CSF2Rβ expression in alveolar macrophages, diminishing cellular responsiveness to CSF2 and impairing phagocytic clearance of pathogens, thereby predisposing the host to secondary pulmonary infections [42]. Similarly, the Orf virus expresses a competitive decoy receptor known as GIF (CSF2 inhibitory factor), which binds with high affinity to ovine CSF2 and IL‑2, inhibiting CSF2/CSF2R interaction and dampening the recruitment and activation of macrophages and DCs during infection [43, 44]. Moreover, the envelope glycoprotein CD2v of African swine fever virus interacted with the CSF2Rα subunit, activated the JAK2 pathway and phosphorylated STAT3, thereby inhibiting apoptosis and promoting viral replication during the early stages of infection [45]. Notably, although exogenous KCR elicited measurable functional responses, its contribution to parasite fitness and pathogenesis could be tested by parasite genetic manipulation such as KCR knockout or conditional depletion and complemented lines followed by infection experiments assessing parasite burden, cytokine milieu, and disease outcomes in our further studies.

In summary, we revealed that KCR engaged host CSF2Rα and modulated the downstream JAK2/STAT5 pathway, thereby regulating macrophage ROS production and phagocytosis. By competitively attenuating host CSF2 responses, KCR supported a model in which KCR acted as a ligand‐like mimic to reshape CSF2 signaling during *T. gondii* infection. These findings expand the understanding of how *T. gondii* manipulates host cytokine networks and highlight the CSF2/CSF2R axis as a potentially important interface for both therapeutic targeting and mechanistic dissection of toxoplasmosis immunopathogenesis.

## Author Contributions

Mingmin Lu conceptualized and designed the experiments and revised the manuscript. Jie Mei performed the experiments and analyzed the statistical data. Xianglin Pu drafted the manuscript. Mengkai Zhang and Yue Liu assisted the experiments and participated in statistical data analysis. Chuang Meng, Lixin Xu, Xiaokai Song, Ruofeng Yan, and Xiangrui Li supervised the experiments implementation and provided the guidance and support.

## Funding

This research was funded by the National Key Research and Development Program of China (Grant 2022YFD1800200), the Jiangsu Provincial Science and Technology Plan Special Fund (Key Research and Development Program‐Social Development, Grant BE2023823), the Fundamental Research Funds for the Central Universities (Grant YDZX2026009), and the Open Project Program of Jiangsu Key Laboratory of Zoonosis (GrantR2506).

## Ethics Statement

All animal procedures were reviewed and approved by the Experimental Animal Welfare and Ethics Committee of the Laboratory Animal Center, Nanjing Agricultural University, China, and were conducted in accordance with the guidelines of the committee (Protocol Number NJAU.No20230911131).

## Conflicts of Interest

The authors declare no conflicts of interest.

## Supporting Information

Additional supporting information can be found online in the Supporting Information section.

## Supporting information


**Supporting Information 1** Table S1: Primers for gene amplification. Table S2: Primer sequences used for the quantitative real‐time PCR

**Supporting Information 2** PCR products of T. gondii KCR and murine CSF2Rα.

## Data Availability

All data generated or analyzed in this research are included in this paper and its additional information files.
