# Supplementary material for: Toxoplasma gondii KCR is a Noncanonical Modulator of CSF2 Signaling that Targets the CSF2Rα–JAK2/STAT5 Axis
Source: Transbound Emerg Dis. 2026 May 9;2026:8426765. doi: 10.1155/tbed/8426765 (PMC13157305; doi:10.1155/tbed/8426765)
Supplement: Supplementary file 1 — Supporting Information 1 Table S1: Primers for gene amplification. Table S2: Primer sequences used for the quantitative real‐time PCR [file TBED-2026-8426765-s001.docx]

**Supplementary file 1: Table 1. Primers for gene amplification**

| **Gene** | **Plasmid** | **Primer sequence (5'→3')** |
| --- | --- | --- |
| KCR | pcDNA3.1 | TAGCGTTTAAACTTAAGCTTGCCACCATGGATCTCTTCAGTTGCGGG |
|  |  | TCGTCCTTGTAATCGAATTCCATGTCCTTCTTAGCCTCCGC |
| CSF2Rα | pCAGGS | ATCATTTTGGCAAAGAATTCATGACGTCATCACATGCCATGA |
|  |  | TGATGGTGATGATGCTCGAGGGGCTGCAGGAGGTCCTTC |

**Supplementary file 1: Table 2. Primer sequences used for the quantitative real-time PCR**

| **RNA target** | **Primer sequence (5'→3')** |
| --- | --- |
| GAPDH | CATCACTGCCACCCAGAAGACTG |
|  | ATGCCAGTGAGCTTCCCGTTCAG |
| CYBB | GGGAACTGGGCTGTGAATGA |
|  | CAGTGCTGACCCAAGGAGTT |
| CYBA | ATGGAGCGATGGTTGTCGG |
|  | TTGATGGTGCCTCCAACCTG |
| NCF1 | TGGAGGGCAGAGACAATCCA |
|  | GACGTCAGCTTCCGTTTGGT |
| NCF2 | CGTCCAAATGAGAGGCAGGT |
|  | GGTTTTGGGTCTGGGAGGAG |
| CR3 | CCACACTAGCATCAAGGGCA |
|  | AAGGGACACACTGACACCTG |
| CD16 | GCTTTTGCAGACAGGCAGAG |
|  | TCCCTTCGCACATCAGTGTC |
| CD64 | AGCGATGGCGTGTATGAAGA |
|  | GGGAAGTTTGTGCCCCAGTA |
| MSR1 | ACAGTTCGACTGGTTGGTGG |
|  | CCTAGACTCCGGCAGACAAC |
| MARCO | GACAAGCCCTTCTTCTCGCT |
|  | AGTTGCTCCTGGCTGGTATG |
