# Supplementary figures and images for: Toxoplasma gondii KCR is a Noncanonical Modulator of CSF2 Signaling that Targets the CSF2Rα–JAK2/STAT5 Axis
Source: Transbound Emerg Dis. 2026 May 9;2026:8426765. doi: 10.1155/tbed/8426765 (PMC13157305; doi:10.1155/tbed/8426765)

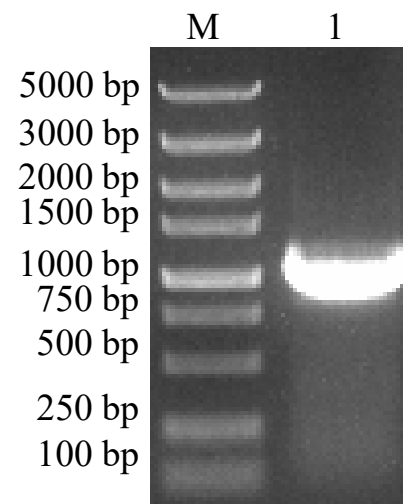

*KCR*

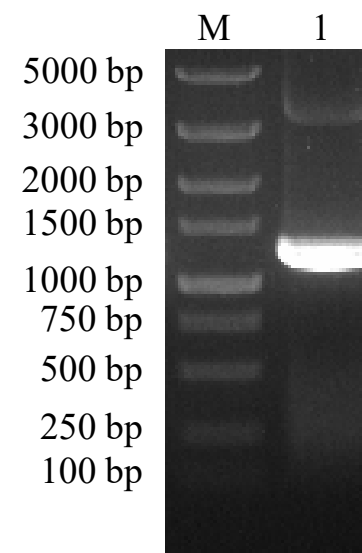

*CSF2Rα*

Supplement: Supplementary file 2 — Supporting Information 2 PCR products of T. gondii KCR and murine CSF2Rα. [file TBED-2026-8426765-s002.pdf]
